# Gut dysbiosis in oncology: a risk factor for immunoresistance

**DOI:** 10.1038/s41422-025-01212-6

**Published:** 2026-01-14

**Authors:** Andrew Allan Almonte, Simon Thomas, Valerio Iebba, Guido Kroemer, Lisa Derosa, Laurence Zitvogel

**Affiliations:** 1 Tumor Immunology and Cancer Immunotherapy, Université Paris-Saclay, Gustave Roussy, Inserm UMR1015, Villejuif, France; 2 Department of Medical, Surgical and Health Sciences, University of Trieste, Trieste, Italy; 3 Université Paris Cité, Sorbonne Université, Inserm, Centre de Recherche des Cordeliers, Paris, France; 4 Centre de Recherche des Cordeliers, Equipe labellisée par la Ligue contre le Cancer, Institut Universitaire de France, Paris, France; 5 Metabolomics and Cell Biology Platforms, Institut Gustave Roussy, Université Paris-Saclay, INSERM US23/CNRS UAR 3655, Villejuif, France; 6 Institut du Cancer Paris CARPEM, Department of Biology, Hôpital Européen Georges Pompidou, AP-HP, Paris, France; 7 Gustave Roussy, Department of Medical Oncology, Villejuif, France

**Keywords:** Tumour immunology, Tumour immunology

## Abstract

The gut microbiome is recognized as a determinant of response to immune checkpoint inhibitor (ICI) therapies in cancer. However, the clinical translation of microbiome science has been hampered by inconsistent definitions of dysbiosis, inadequate biomarker frameworks, and limited mechanistic understanding. In this review, we synthesize the current state of knowledge on how gut microbial composition and function influence ICI efficacy, highlighting both correlative and causal evidence. We discuss computational approaches based on α-diversity or taxonomic abundance and argue for more functionally and clinically informative models, such as the topological score (TOPOSCORE) and other dysbiosis indices derived from machine learning. Using retrospective analyses of metagenomic datasets from thousands of patients and healthy controls, we examine microbial patterns that distinguish responders from non-responders. We also explore how dysbiosis perturbs immunoregulatory pathways, including bile acid metabolism, gut permeability, and mucosal immunomodulation. Finally, we assess emerging therapeutic strategies aimed at correcting microbiome dysfunction — including dietary modification, bacterial consortia, and fecal microbiota transplantation — and describe how they are being deployed in multiple clinical trials. We conclude with a brief discussion of the ONCOBIOME initiative, which works with international partners to incorporate microbiome science into oncology workflows. By refining our understanding of gut–immune interactions and translating it into action, microbiome-informed oncology may unlock new therapeutic potential for patients previously resistant to immunotherapy.

## Introduction

Intestinal dysbiosis, broadly defined as an imbalance in the composition or function of the gut microbiota, is a significant contributor to resistance against immune checkpoint inhibitor (ICI) therapies.^1–16, ^ However, despite the significant resources used to map the human gut microbiome through the Human Microbiome Project and bring clarity to these enteric states,^17–19^ there is still no consensus definition of what constitutes a “healthy” (eubiotic) vs a dysbiotic microbiome, complicating translation into biomarkers and therapeutics. Clarifying and operationalizing these concepts is critical for improving patient stratification and the rational use of microbiota-centered interventions (MCIs), such as dietary regimens and fecal microbiota transplantation (FMT), to overcome ICI resistance.

Skepticism about the terms “dysbiosis” and “eubiosis” persists, with critics arguing that they are imprecise or pre-scientific.^20–22^ Nonetheless, in oncology settings, dysbiosis can be measured and correlated with ICI outcomes, and is amenable to intervention.^23^ Key metabolic and genomic markers have been proposed and validated in different disease contexts, and more are being investigated. This has led to the realization that dysbiosis may not be a uniform condition; our group has identified at least two distinct forms of dysbiosis, defined by either the close-to-complete absence or overabundance of *Akkermansia muciniphila*.^3^ This is notable because *A. muciniphila* has been previously linked to improved ICI responses.^13^ Indeed, this trichotomic observation indicates a more complicated, non-linear relationship with anti-tumor immunity.^24^

In this review, we examine the evolving conceptual frameworks for defining and measuring dysbiosis in the context of ICI therapy. We outline key factors that influence gut microbial health and that need to be considered when attempting to define a “healthy” vs “imbalanced” gut microbial community. We then assess the current toolbox available for detecting and quantifying dysbiosis, ranging from serum and fecal biomarkers to classical diversity metrics and taxonomic signatures, to newer approaches that integrate ecological topology and functional inference. Finally, we discuss how dysbiosis metrics might be translated into clinical practice as biomarkers of response, guides for patient stratification, and rationale for implementing MCIs.

## Defining a healthy microbiome

The World Health Organization constitution defines health as “a state of complete physical, mental, and social well-being and not merely the absence of disease or infirmity”.^25^ This definition has been elaborated upon by identifying and describing eight key interconnected hallmarks of health within three broader categories: spatial organization (maintaining barrier integrity and containing insults); homeostasis (healthy turnover, circuit integration, and rhythmic oscillations); and responses to stress (resilience, repair, and hormesis).^26^

**Fig. 1:**
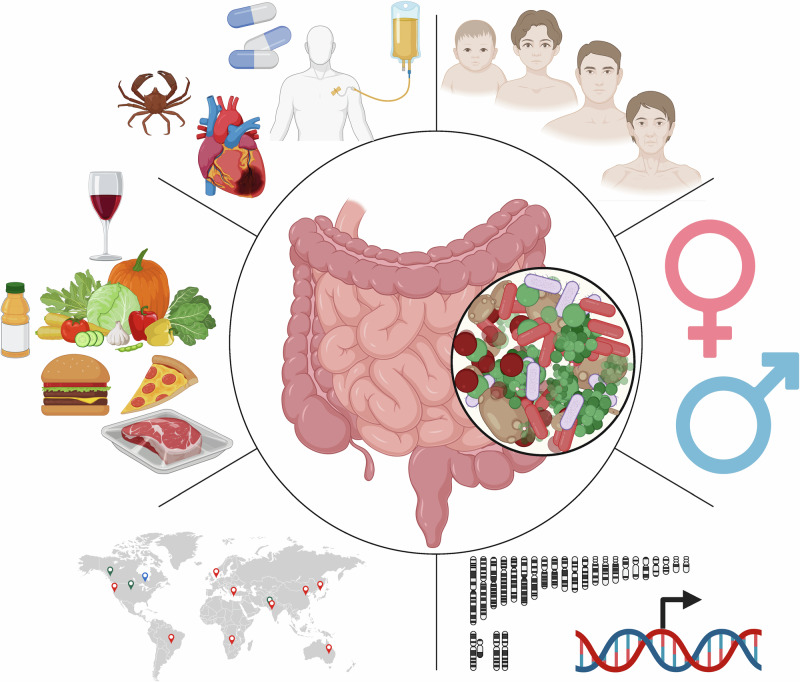
Multilayered determinants of gut-microbiota ecology. A schematic overview of the principal host-intrinsic and environmental factors known to shape intestinal microbial composition, diversity, and function. Clockwise from top: biological age; sex and sex hormones; host genetics; geography and ethnicity; dietary patterns; chronic diseases; and medications and co-medications, including antibiotics, proton-pump inhibitors, and benzodiazepines. These factors interact to modulate microbial community structure, SCFA production, bile acid metabolism, and immune tone, thereby influencing health trajectories and the efficacy of ICIs. Illustration created with BioRender.com.

Applying and integrating this holistic view to the gut ecosystem is challenging because a “healthy” or eubiotic microbiome may entail more than simply the absence of dysbiosis or the presence of a few keystone taxa, but likely results from the dynamic interplay of these factors. Indeed, inherent host factors such as age, gender, or genetics can influence the gut microbiota composition, but external factors such as diet, medication, and lifestyle also contribute to continually shaping microbiota composition. Any practical definition must therefore consider both the intrinsic and extrinsic factors that shape microbial communities across an individual’s lifespan (Fig. 1).

### Age and microbiome trajectories

Biological age is one of the strongest determinants of gut microbiota composition.^27^ Across a person’s lifespan, three broad successional phases can be distinguished. Primary succession involves rapid assembly of a community — set in utero and at delivery — that settles into a relatively stable consortium dominated by Firmicutes and Bacteroidetes.^28,29^ As pregnancy advances, the vaginal microbiota diversifies,^30^ and delivery provides a critical first inoculum for the infant gut. Vaginally delivered infants acquire maternal Bacteroides, whereas Cesarean birth favors early colonization by *Enterococcus*, *Enterobacter*, and *Klebsiella* pathobionts.^27^ Cesarean delivery also predicts a more unstable neonatal microbiome and a heightened risk of infections, immune disorders, obesity, and neuroendocrine disturbances.^31–33^ Breastfeeding tempers these effects by delivering secretory immunoglobulin A (IgA), antimicrobial peptides, and prebiotic human milk oligosaccharides, which foster *Bifidobacterium* dominance and direct the orderly succession from pioneer taxa to a toddler-age consortium rich in *Bifidobacterium*, *Clostridium*, and *Bacteroides*.^34^ By 3–6 years, the microbiota approaches an adult-like “climax” community that remains comparatively stable thereafter.

Secondary succession in early to mid-adulthood reflects dietary diversification, lifestyle factors, and environmental exposures. However, day-to-day oscillations persist. Circadian cues shift the abundance of *Clostridiales*, *Lactobacillaceae*, and *Bacteroidales* in mice, and ~10% of human operational taxonomic units display similar time-of-day variation.^35,36^ Psychosocial stress likewise skews the community, increasing *Clostridium* and depleting *Bacteroides*.^37^ Despite such perturbations, adult microbiomes co-evolve with their hosts, as in situ bacterial genome adaptation fosters long-term colonization.^38^

Finally, late life ushers in tertiary succession, which is marked by declining α- and β-diversity and loss of keystone commensals like *Faecalibacterium* and *Bifidobacterium*.^39,40^ Healthy ageing is associated with butyrate-producing taxa, an expanded *Bacteroides* enterotype (discussed below), and enrichment of *Alistipes* spp. or *A. muciniphila*, which generates anti-inflammatory polyamines and bile acid (BA) derivatives.^41,42^ By contrast, frailty and multimorbidity are correlated with blooms of *Enterocloster*, *Eggerthella*, and other opportunists,^43^ which can be associated with a dysbiotic gut.

### Sex and the gut microbiome

Women generally harbor richer and more diverse intestinal communities than men.^44–46^ They are relatively enriched in *Alistipes* spp., *A. muciniphila*, *Clostridium symbiosum*, and butyrate-biosynthesis genes, whereas men carry more *Prevotella* spp. and BA transporter genes.^44,45^ The endocrine milieu helps explain these patterns. For example, hormonal contraception and menstrual status shift microbial profiles in women.^44^ Moreover, a meta-analysis of 13 studies and 10,468 samples found that higher circulating estrogen was correlated with increased microbial diversity, greater *Bacteroides* abundance, and lower *Ruminococcaceae* abundance, whereas testosterone levels were correlated with *Ruminococcus*, *Acinetobacter*, and overall diversity in men.^47^ Menopause further reshapes the community: premenopausal women display higher Firmicutes-to-Bacteroidetes ratios and higher levels of *Roseburia*, whereas the post-menopausal decline in estrogen is accompanied by lower diversity and expansions of taxa linked to cardiometabolic risk.^48–50^

Whether this variation is enough to cause differential responses to ICIs between men and women is not understood. If present, the influence of sex-specific microbial variation would be very small. The total variance in gut microbial composition attributable to sex is modest (~0.5%) but biologically meaningful alongside stronger drivers such as diet or medication.^44^ Some studies have investigated whether sex-specific variations influence a patient’s outcome following ICI therapy. Two meta-analyses of 20 and 14 randomized clinical trials, involving more than 15,000 patients diagnosed with different indications, found that although ICIs enhance anti-cancer responses in both men and women, men appear to experience a modestly greater benefit.^51,52^ However, the generalizability of these findings has been debated^53,54^ owing to some inconsistencies between studies.^55–57^

### Host genetics and the microbiome

Host genetics leaves a discernible, though limited, imprint on gut microbial composition. Genome-wide analyses estimate that host variants account for roughly 2%–8% of interindividual compositional variation,^58,59^ and a cohort of over 1500 healthy adults revealed that nearly one-third of fecal taxa are heritable.^60^ Notably, the most robust gene–microbe associations cluster around loci that govern diet, metabolism, immunity, and mucosal glycosylation. Lactase-persistence alleles at the *LCT* gene consistently track with higher abundances of *Bifidobacterium* spp.,^61,62^ whereas polymorphisms in the *FUT2/FUT1* secretor locus and variants near the *ABO* gene modulate fucosylated mucin production and favor mucin specialists like *Ruminococcus torques*.^63,64^ Twin studies extend this picture, showing that highly heritable taxa often co-segregate with alleles involved in barrier defense, olfaction, and short-chain fatty acid (SCFA) metabolism. For example, *Christensenella* spp. is associated with lean body mass and variants in genes such as *ALDH1L1* and *GNA12*.^65,66^ Although genotype shapes the niche space for specific microbes, environmental exposures, lifestyle, and stochastic colonization dominate the wider landscape of the adult gut ecosystem.

### Geography and lifestyle

Geography and ethnicity shape gut microbial composition, but their influence is channeled largely through diet and lifestyle. Comparative surveys show that the fiber-rich, agrarian diets typical of rural Africa promote Bacteroidetes-enriched, *Prevotella*-dominated communities that maximize fermentation and suppress inflammatory pathobionts, whereas western cohorts — characterized by higher fat and lower fiber intake — display reduced α-diversity, depletion of *Prevotella*, and expansion of Enterobacteriaceae like *Escherichia* and *Shigella*.^67,68^ Even within a single continent, country-specific differences persist; ethnicity alone accounts for about 6% of interindividual variance, and migration studies show that acculturation rapidly shifts the microbiome toward the host country’s profile.^69–72^ Lifestyle compounds these effects: regular physical activity enhances diversity, enriches SCFA producers, and correlates with favorable cardiometabolic markers.^70,73–75^ This is particularly salient for cancer patients undergoing ICI therapy, as exercise has been demonstrated to modulate gut microbial composition and correlate with increased levels of formate, which enhances the cytotoxic activity of CD8^+^ T-cells.^10^ Geography, ethnicity, and lifestyle intersect to generate distinct, yet flexible, microbial landscapes.

### Dietary influences

Diet is the most malleable — and most powerful — determinant of gut microbiota composition. Poor diets now surpass other behavioral risk factors for global mortality,^76^ and controlled feeding studies show that the gut microbiota can shift within days of a dietary change.^77^ Western-style, high-fat, animal-based diets promote bile-tolerant, pro-inflammatory taxa (e.g., *Alistipes*, *Bilophila wadsworthia*, *R. torques*), reduce diversity, deplete *Prevotella*, and expand *Proteobacteria*, driving barrier dysfunction, endotoxemia, and chronic inflammation.^77–80^ Fiber-rich, plant-based diets have the opposite effect, nurturing *Bifidobacterium* and *Lactobacillus*, increasing butyrate producers like *Roseburia* and *Eubacterium rectale*, and shielding against obesity and insulin resistance via SCFA signaling.^15,70,81–83^ Very-low-carbohydrate ketogenic regimens selectively expand mucin specialists like *A. muciniphila* and *Roseburia intestinalis*,^84^ whereas Mediterranean diets — abundant in vegetables, legumes, fruits, fish, and olive oil — increase *Faecalibacterium prausnitzii*, *Bacteroides cellulosilyticus*, and *Roseburia* spp., lower secondary BAs, attenuate systemic inflammation, and correlate with reduced risks of disease.^70,85,86^

Diet continually shapes the gut ecosystem, with downstream consequences for immune tone, metabolic health, and disease trajectory. Dietary modulation has therefore been proposed as a strategy to enhance ICI efficacy and is arguably the least intrusive MCI.^15,87^ Several clinical trials are currently underway to determine its potential to improve cancer immunotherapies. We have recently published a tabulated list of current MCI trials and encourage readers to consult it for further insights.^88^

### Medications and co-medications

Medications rival diet as modulators of gut ecology. Together, they explain nearly 20% of interindividual variation in microbial composition, with drugs often exerting the stronger influence.^59,70^ Broad surveys identify antibiotics,^16^ proton-pump inhibitors (PPIs),^89^ benzodiazepine derivatives,^90^ antidiabetics, non-steroidal anti-inflammatories, and immunosuppressants as major drivers of microbial richness, diversity, and composition.^43,58,70,91^ PPIs, for instance, enrich Firmicutes and oral taxa like *Streptococcus salivarius* and *Micrococcaceae*, expand *Veillonella* and *Enterocloster*, and reduce overall diversity, a profile linked to *Clostridioides difficile* infection and adverse metabolic shifts.^43,92–97^ Antibiotics predictably inflict the most acute damage, reducing diversity, depleting beneficial commensals, and permitting blooms of pathobionts like *Enterocloster* and *Streptococcus*.^4,27,98^ Indeed, long-term use of these medications can leave persistent alterations in the gut microbiome that can endure years beyond treatment discontinuation.^99^

High-throughput transcriptomics and machine learning (ML) approaches can now map these drug–microbe interactions in finer detail. Bacteroides appear particularly susceptible to non-antibiotic toxicities, and agents like simvastatin can downregulate bacterial riboflavin biosynthesis.^100^ In silico models further reveal that lipophilicity and charge distribution best predict a molecule’s anti-commensal potency, with PPIs, immunosuppressants, and antineoplastic drugs ranking among the most dysbiotic.^101^ Polypharmacy amplifies these effects in older adults, eroding biodiversity, curtailing SCFA production, and fostering expansions of *Streptococcus* and *Lactobacillus* spp.^102,103^

Several large-scale meta-analyses have demonstrated that these iatrogenic effects have practical clinical implications. A meta-analysis of 105 studies and 46,000 cancer patients treated with ICIs convincingly showed that exposure to antibiotics was associated with shorter progression-free survival and overall survival in NSCLC patients.^104^ Another meta-analysis that examined 41 studies and 20,000 patients similarly demonstrated the negative impact of PPI treatment on patients treated with ICIs.^105^ Benzodiazepine has also been shown to compromise immunotherapy, with preclinical and clinical data demonstrating that these agents suppress anti-tumor immune responses and correlate with reduced survival in NSCLC patients receiving programmed death protein-1 (PD-1) blockade.^90,106^ These large-scale studies demonstrate that medication-induced dysbiosis has a negative impact on the outcome of patients treated by immunotherapy. Drug exposure, therefore, represents a risk factor for a dysbiotic microbiota.

### Diseases

Disease states almost invariably influence the gut ecosystem, and, in many conditions, microbial shifts appear to participate in pathogenesis. Metabolic disorders illustrate this reciprocity: obesity is correlated with an elevated Firmicutes-to-Bacteroidetes ratio and blooms of *Roseburia faecis* and *Ruminococcus gnavus*;^43,107^ non-alcoholic liver dysfunction is associated with diminished *E. rectale* and higher circulating secondary BAs;^108^ and type 2 diabetes features expansions of Enterobacteriaceae, *Escherichia coli*, *Bacteroides caccae*, and *Prevotella copri*.^109^ Inflammatory bowel disease (IBD) is marked by surges of pro-inflammatory Veillonellaceae and Proteobacteria and a concomitant loss of SCFA producers like *Ruminococcus bromii*, *Bifidobacterium* spp., and *F. prausnitzii*.^110^ Cardiovascular pathology is likewise linked to microbial signatures, as atherosclerosis is accompanied by increased *Streptococcus*, *Escherichia*, and *Shigella* spp.^111^

Cancer progression exerts systemic effects that profoundly alter host–microbe interactions in the gut. Murine models of cancer cachexia show a dysbiosis characterized by expansion of *Enterobacteriaceae*^112^ such as *Klebsiella oxytoca*, a gut pathobiont whose growth follows the depletion of butyrate-producing taxa.^113^ These microbial perturbations are associated with elevated systemic interleukin-6 (IL-6) and lipopolysaccharide-binding protein (LBP), biomarkers predictive of cachexia and poor survival in colorectal and lung cancer.^112^ Murine studies have also demonstrated that tumor growth can induce a β-adrenergic receptor (βAR)-dependent “stress ileopathy” marked by villous atrophy, epithelial permeability, and a persistent *Clostridium* bloom at the expense of *Lactobacillus*.^114^ Tumor progression has also been associated with a reduction in fecal and systemic tryptophan — an essential amino acid and microbial substrate. Restoring tryptophan, either through dietary supplementation or colonization with *Duncaniella dubosii*, appears to re-establish immune balance and support antitumor T-cell activity.^115^ Similar patterns emerge in pediatric neuroblastoma patients: reduced richness, depletion of butyrate producers, and enrichment of proteolytic fermenters define a conserved “oncomicrobiome” signature.^116^

This dysbiotic state has direct consequences for ICI efficacy. Chronic activation of the sympathetic axis through βAR signaling suppresses cytotoxic T-cell function, whereas β-blockade restores effector activity and improves survival in patients receiving immunotherapy.^117^ Sustained adrenergic signaling also shapes microbial ecology, promoting *Clostridium*-dominated dysbiosis that blunts anti-PD-1 responses.^114^ Conversely, tryptophan supplementation or *D. dubosii* colonization in glioblastoma models reinstates CD8^+^ T-cell circulation from the bone marrow to the tumor bed, enhancing ICI efficacy.^115^ This neuro-immune-microbial axis, linking stress, dysbiosis, and immune suppression, provides a mechanistic continuum from barrier disruption to therapeutic resistance. Historical pathology foreshadowed this interplay: in 1965, Wangel and Deller described villous atrophy and steatorrhoea in a patient with bronchial carcinoma, attributing it to a tumor-derived systemic factor.^118^ The convergence of such early observations with contemporary mechanistic insights reinforces the notion that any operational definition of a “healthy” microbiome must account for distant (extraintestinal) disease states.

## Serum and fecal markers of dysbiosis

**Fig. 2:**
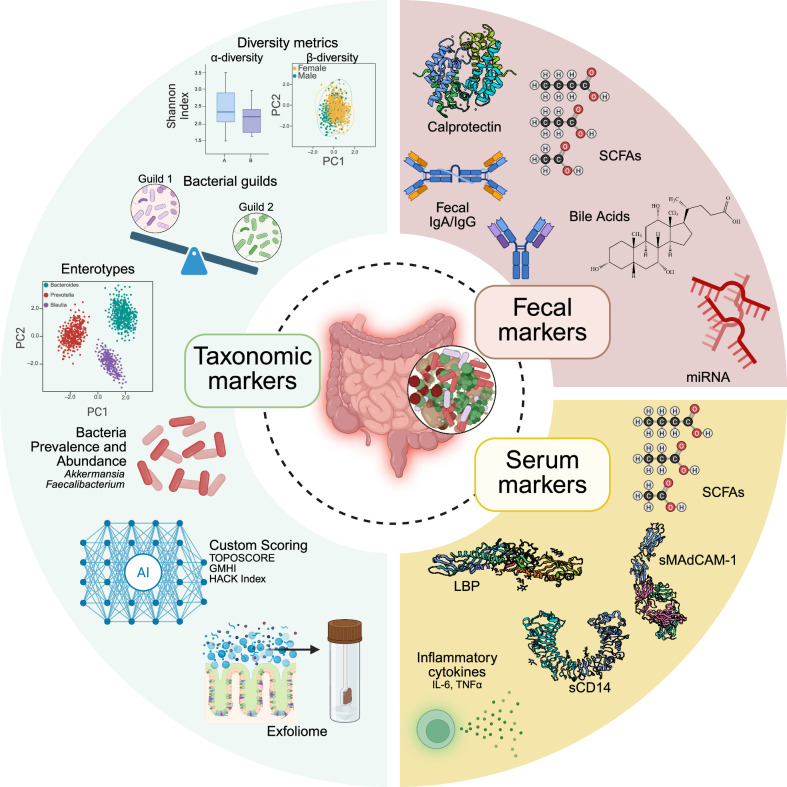
Multi-omic framework for detection of intestinal dysbiosis. The dysbiotic gut is at the center of three complementary biomarker domains. Taxonomic markers (green) encompass ecological read-outs — α- and β-diversity, enterotype and bacterial-guild stratifications, prevalence/abundance of signature taxa such as *Akkermansia* and *Faecalibacterium*, and AI-enabled composite scores. Emerging “exfoliome” assays also capture host and microbial material shed from the gut epithelium, providing additional insight into enteric health. Fecal markers (pink) quantify host-derived inflammation (calprotectin), mucosal immune activity (fecal IgG and IgA), metabolic outputs of microbiota such as SCFAs and BAs, and regulatory miRNAs. Serum markers (yellow) extend surveillance systemically, measuring translocated microbial metabolites, classic “leaky gut” markers, endothelial activity (sMAdCAM-1), and pro-inflammatory cytokines. Collectively, these markers enable a holistic, compartmentalized assessment of dysbiosis. Three-dimensional protein structures were obtained from the Protein Data Bank, and the figure was created with BioRender.com.

In oncology, some of the most clinically tractable indicators of dysbiosis are metabolites and host-derived proteins measurable in stool or blood. These biomarkers capture functional consequences of microbial imbalance — such as mucosal inflammation, barrier disruption, and altered metabolism — and several have been linked to cancer progression and response to therapy. Although a precise definition of dysbiosis remains elusive, combining systemic, fecal, and taxonomic markers (Fig. 2) enables researchers to assess microbial diversity, abundance, and activity in ways that can provide insight into the microbiome’s influence on immunotherapy outcomes. In the following section, we discuss some of the systemic and fecal biomarkers that can be used to diagnose dysbiosis by routine serological or stool assays.

### Fecal biomarkers

Dysbiosis often manifests locally as intestinal inflammation and disrupted barrier function, which can release host proteins into stool. One classical example is fecal calprotectin, a calcium-binding heterodimer (S100A8/S100A9) from neutrophils that is elevated in the stool during gut inflammation (Fig. 2).^119,120^ It is an established, sensitive marker used to monitor IBD activity and, by extension, reflects microbiota-driven mucosal inflammation.^119,121,122^ Elevated fecal calprotectin has been correlated with dysbiotic alterations in microbiota composition among IBD patients,^123^ highlighting that shifts in commensal populations can incite neutrophilic inflammation. Fecal lactoferrin and lipocalin-2 are additional neutrophil-derived proteins detectable in stool and are well-validated proxies for gut inflammation and barrier stress in IBD.^124,125^ Although these stool inflammatory markers were originally developed in the context of IBD, they can be translatable to other contexts of dysbiosis (e.g., cancer) as non-invasive proxies for microbiota-driven mucosal immune activation.

Microbiota-derived metabolites in stool are also being studied as functional dysbiosis markers in oncology. SCFAs are key immunomodulatory microbial metabolites produced by fermentative commensals (e.g., *Faecalibacterium* and *Roseburia* spp.). They signal through G protein-coupled receptors to promote anti-inflammatory regulatory T-cells and enhance CD8^+^ T-cell effector functions.^126^ Butyrate is a frequently studied SCFA compound that has been shown to boost anti-tumor immunity in preclinical models.^127^ Patients with dysbiosis often have a reduced abundance of butyrate producers and lower overall SCFA levels due to a loss of beneficial fermenters, and their depletion in dysbiosis could thus signal an impaired immunological tone. Clinically, measurement of fecal SCFAs or their ratios is an emerging approach for functional characterization of dysbiosis in diseases like IBD and colorectal cancer (CRC).^128,129^

**Fig. 3:**
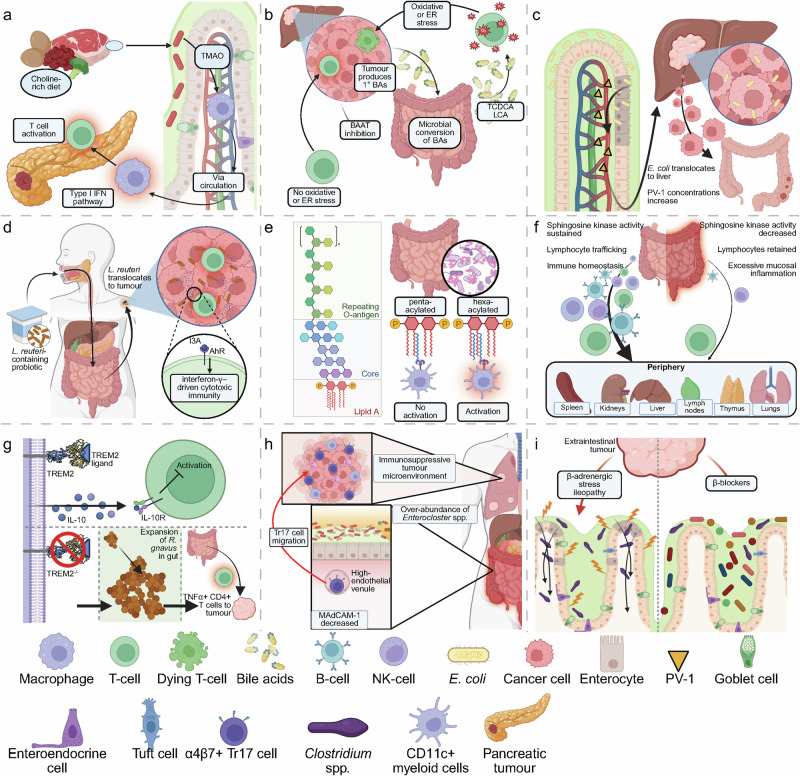
Integrated overview of key mechanisms that link the gut microbiota, immune cell trafficking, and tumor immunity across landmark studies. These studies examine distinct yet interconnected pathways linking the gut and tumors, including metabolite recirculation (**a**, **b**), bacterial translocation (**c**–**e**), immune cell trafficking (**f**–**h**), and neurotransmitter-mediated stress responses (**i**). **a** A choline-rich diet enhances microbial production of trimethylamine, which is converted to TMAO.^160^ Circulating TMAO activates macrophages through the type I IFN pathway, reprogramming the tumor microenvironment in pancreatic cancer to support effector CD8^+^ T-cell activation and responsiveness to checkpoint inhibitors. Dietary choline supplementation or TMAO treatment reduces tumor burden and restores immunotherapy sensitivity. **b** In hepatocellular carcinoma, upregulated BA synthesis and bile acid–CoA:amino acid *N*-acyltransferase (BAAT)-mediated conjugation drive the accumulation of conjugated primary BAs (e.g., taurochenodeoxycholic acid (TCDCA)) and secondary species (e.g., lithocholic acid (LCA)).^240^ These metabolites suppress antitumor CD8^+^ T-cell function, with TCDCA causing oxidative stress and LCA inducing endoplasmic reticulum (ER) stress. BAAT inhibition restores T-cell activity and sensitizes tumors to PD-1 blockade. **c** Compromise of the gut vascular barrier (GVB) allows *E. coli* to translocate to the liver, where they establish a premetastatic niche, promoting CRC metastasis.^241^ The endothelial marker PV-1 serves as a biomarker for GVB disruption and predicts metastatic spread. **d** The commensal *Lactobacillus reuteri* can translocate into tumors and metabolize dietary tryptophan into indole-3-aldehyde (I3A).^1^ I3A activates the aryl hydrocarbon receptor (AhR) in CD8^+^ T-cells, stimulating IFN-γ-driven cytotoxic immunity and potentiating ICI responses. **e** Acylation pattern of bacterial LPSs determines their immunostimulatory potential. Hexa-acylated LPS from specific gut commensals potently activates the host immune system via TLR4, promoting antitumor CD8^+^ T-cell responses and improving the efficacy of anti-PD-1 therapy.^135^ By contrast, penta-acylated LPS species fail to activate immunity and are correlated with resistance to checkpoint blockade. **f** Kaede photoconvertible mice were used to map intestinal immune cell migration, showing continuous egress of B, T, and innate lymphoid cells from the colon to peripheral organs through a sphingosine-1-phosphate (S1P)-dependent pathway.^242^ Under eubiosis, sphingosine kinase activity sustains S1P gradients and normal lymphocyte trafficking; dysbiosis or inflammation disrupts this axis, trapping cells in mucosal tissues and altering systemic immune homeostasis. **g** Loss of the lipid-sensing receptor TREM2 (triggering receptor expressed on myeloid cells-2) on intestinal macrophages reprograms them toward a pro-inflammatory state, reducing IL-10 production and expanding *R. gnavus* in the colon.^243^ This microbial shift drives TNF-producing CD4^+^ T cells to migrate from the gut to tumors, amplifying local antitumor responses and improving anti-PD-1 efficacy. **h** Antibiotics or dysbiosis downregulate the adhesion molecule MAdCAM-1 in the ileum, allowing α4β7^+^RORγt^+^ Treg17 (Tr17) cells to migrate into tumors and suppress immunity.^4^ This process, driven by *Enterocloster* species and BA signaling, undermines anti-PD-1 efficacy. **i** Extraintestinal tumors induce a β-adrenergic-driven stress ileopathy that damages the ileal mucosa, increasing permeability and enabling dysbiosis dominated by *Clostridium* species.^114^ This fosters tumor progression, whereas β-blockers or vancomycin restore barrier integrity, limit microbial overgrowth, and slow cancer growth. Figure was created using BioRender.com.

SCFAs are not the only fecal metabolites being investigated in a cancer context. Abnormal profiles of BAs in stool can also reflect dysbiosis. Gut microbes convert host BAs into secondary BAs, and a dysbiotic microbiome can skew this conversion, resulting in excess production of immunosuppressive BA species that may contribute to disease (Fig. 3b).^130^ For instance, our lab has shown that antibiotic-induced dysbiosis spurs the downregulation of mucosal vascular addressin cell adhesion molecule 1 (MAdCAM-1; discussed below) via altered BA metabolism (Fig. 3h).^4^ Fecal BA composition is therefore being investigated as a proxy for dysbiosis. Another less conventional fecal dysbiosis marker is the fecal microRNA (miRNA) profile. The gut can shed host miRNAs into the stool, and recent studies indicate that they might reflect the state of the gut mucosa and microbiome.^131^ Although not yet used clinically, stool miRNA panels are a novel dimension of the “exfoliome” of the gut epithelium that could furnish indirect readouts of microbiome status in disease.^24,132^

Collectively, these fecal markers represent neutrophil-driven mucosal inflammation, while metabolite profiles provide a window into microbial metabolic capacity. Together, they demonstrate that dysbiosis is not defined by any single marker but by the convergence of immune, barrier, and metabolic perturbations.

### Systemic biomarkers

Because gut dysbiosis and cancer^114^ have been associated with a compromised intestinal barrier, markers of intestinal permeability and microbial translocation are of great interest. Zonulin, a regulator of gut permeability, is released when barrier integrity is compromised; elevated serum zonulin has therefore been investigated as a proxy for “leaky gut” in cancer patients.^133^ Other systemic indicators of epithelial injury include intestinal fatty acid-binding protein, which reflects enterocyte damage, and circulating citrulline, a marker of functional mucosal surface area.^87^ Together, these markers provide clinically accessible readouts of barrier dysfunction that may help stratify patients at risk of dysbiosis-associated inflammation and poor therapeutic response. Perhaps the most widely studied blood markers of dysbiosis are those that reflect endotoxin leakage from the gut. An outgrowth of Gram-negative bacteria leads to excess lipopolysaccharide (LPS) in the gut lumen, which can translocate into the bloodstream if the mucosal barrier is compromised.

Notably, bacteria can present diverse variations of the LPS molecule, each with distinct chemical, structural, and functional heterogeneity and varying immunogenicity (Fig. 3e).^134^ These factors can influence immunotherapy outcomes. For example, the number of acyl chains attached to lipid A — the most conserved and immunologically active portion of LPS — can modulate host toll-like receptor 4 (TLR4) signaling and thereby influence the efficacy of anti-PD-1 immunotherapy.^135^ One *A. muciniphila* strain isolated from a healthy donor produces an LPS with a lipid A moiety that beneficially tunes innate immune signaling and restores intestinal and metabolic homeostasis.^136^ LPS of *A. muciniphila* also lacks the O-antigen (the most variable and accessible portion of LPS^134^), which results in the induction of IL-10 expression and consequent anti-inflammatory effects.^137^ The O-antigen of *Salmonella* shields the lipid A moiety, delaying or reducing TLR4 recognition and consequently weakening host antibacterial responses.^138^

LPS in the blood triggers an innate immune response and leads to the production of LBP and soluble CD14 (sCD14) to bind and neutralize it (Fig. 2). Hence, high concentrations of circulating LBP and sCD14 are frequently used as indirect markers of microbial translocation and have been linked to inflammatory sequelae in cirrhosis, although their utility as definitive surrogates remains under active investigation.^139^ Western-style high-fat diets, which correlate with dysbiosis, are associated with increased gut permeability and higher serum sCD14 levels.^140^ In metabolic disorders, elevated LBP and sCD14 frequently accompany insulin resistance, thus supporting the concept of “metabolic endotoxemia” driven by dysbiosis.^141,142^ Notably, these markers have clinical prognostic value: one study has linked high baseline LBP and anti-flagellin IgA levels to increased risk of hepatocellular carcinoma in patients with liver disease,^143^ suggesting that gut-derived circulating endotoxin may promote oncogenesis in the liver.

Low-grade, systemic inflammation is a hallmark of unhealthy gut microbial states. Patients with CRC commonly show elevated serum pro-inflammatory cytokines (such as tumor necrosis factor-α (TNF-α) and IL-6) and C-reactive protein alongside high LBP.^87^ These inflammatory mediators are not specific to the gut, but when found in combination with evidence of microbial translocation, they strengthen the case that dysbiosis is driving systemic inflammation. Importantly, interventions that modulate the microbiome can attenuate these markers. For example, supplementation with *A. muciniphila* has been shown to lower circulating LPS, LBP, and inflammatory cytokines in mouse models.^144^ Indeed, *Akkermansia* p2261 is currently being investigated as a live biotherapeutic designed to improve ICI therapy responses (NCT05865730) and has very recently been shown to improve CAR T-cell therapy.^145^ Such findings suggest that correcting dysbiosis not only modifies microbial composition but also dampens systemic inflammatory responses, reinforcing the causal link between gut microbiota and systemic inflammation. Therefore, tracking a panel of LPS-related genes and proteins^135^ and blood cytokines offers a window into the host’s inflammatory response to the gut microbiota.

A variety of small-molecule metabolites produced or modulated by gut microbes serve as indirect markers of dysbiosis in host circulation. SCFAs are again a prominent example and, although mostly assessed in stool, can also be easily measured in serum. Higher systemic butyrate levels generally indicate a fiber-rich diet and healthy microbiota, whereas low SCFAs or an imbalanced SCFA ratio can signal the loss of key beneficial taxa. SCFA levels not only help signal dysbiosis but may actively influence outcomes in diseases like cancer by shaping T-cell responses.^126^ Clinical studies have linked deficient SCFA production to conditions such as graft-versus-host disease and poor responses to cancer immunotherapies, spurring interest in SCFA supplementation or diets to restore microbial balance.^15,77,88,146,147^

However, mouse models have highlighted inconsistencies in treatment outcomes following supplementation with SCFAs. They can enhance PD‑1 blockade when administered orally (drinking water or gavage) at physiologically relevant concentrations.^148–150^ By contrast, supplementing mice with butyrate in their drinking water appears to be detrimental for CTLA‑4 blockade,^151^ suggesting that baseline SCFA levels or their supplementation may differentially affect ICI regimens. Moreover, the route of administration and context matter; systemic butyrate given by intraperitoneal injection improved anti‑PD‑1 efficacy,^127^ whereas intratumoral butyrate may drive tumor fatty acid oxidation and lead to treatment resistance.^152^ Notably, SCFA supplementation without ICI does not improve tumor control or survival.^127^ These results highlight the need for more research in this area before implementing them in clinical practice.

Perhaps the most exciting new serum marker is soluble MAdCAM-1 (sMAdCAM-1). Our lab recently reported that low baseline sMAdCAM-1 levels are strongly associated with gut dysbiosis in cancer patients.^4,24^ In several independent patient cohorts, low sMAdCAM-1 predicted poorer outcomes and was linked to either recent antibiotic use or intrinsic dysbiosis.^4^ Mechanistically, antibiotic-induced dysbiosis leads to the “blooming” of bacteria from the *Enterocloster* genus, which downregulates MAdCAM-1 expression in the gut vasculature owing to altered BA metabolism (Fig. 3h). This, in turn, allows immunosuppressive gut-resident α4β7^+^FoxP3^+^ regulatory Th17 (Tr17) cells to exit the gut and migrate to peripheral tumors, where they can blunt local antitumor immunity.^4^ Conversely, high sMAdCAM-1 indicates an intact gut immune recruitment axis and correlates with richer microbial diversity. Thus, sMAdCAM-1 has been proposed as a “gut check” biomarker for dysbiosis in oncology and ICI therapy responses.^153^ Low levels indicate microbiome imbalance and immune impairment, potentially identifying patients who might benefit from MCIs before or during immunotherapy. Indeed, a recent retrospective validation of the negative prognostic value of lower sMAdCAM-1 has been reported in 612 patients in first- and second-line therapies for metastatic renal cell carcinoma (RCC).^154^

Other gut-derived metabolites can serve as proxies for dysbiosis. Perturbations in microbiota-dependent tryptophan metabolism have been reported in IBD, metabolic syndrome, and Western lifestyles, all contexts associated with microbial imbalance.^155^ Another example is trimethylamine-*N*-oxide (TMAO), a hepatic metabolite generated from gut-derived precursors such as choline and L-carnitine.^156^ Elevated TMAO levels have been linked to cardiovascular disease, type II diabetes, and obesity,^157^ all of which correlate with dysbiosis. Broad-spectrum antibiotic use reduces TMAO production,^158^ further underscoring its microbial origin. However, this effect appears to be context-dependent, as preclinical studies in breast^159^ and pancreatic cancer models^160^ indicate that TMAO may improve responses to ICIs (Fig. 3a). For instance, TMAO and its microbial precursors reprogrammed tumor-associated macrophages toward a pro-inflammatory, type I interferon (IFN)-dependent phenotype, augmenting CD8^+^ T-cell activity and potentiating anti-PD-1 efficacy in murine pancreatic cancer.^160^ These findings demonstrate that microbiota-derived metabolites may either impair or amplify immunotherapy outcomes depending on the metabolic and microbial context.

Taken together, these biomarkers illustrate that dysbiosis leaves measurable systemic and mucosal imprints that can be tracked in oncology cohorts, providing mechanistic links between microbial imbalance, systemic inflammation, and therapy response.

## Simplifying gut microbiome complexity for clinical utility

Given the inherent complexity and variability of gut microbiome data, it is necessary to simplify it into an easily interpretable, more clinically relevant metric. Doing so has several advantages. First, “-omics” data are noisy and high-dimensional, and rarely have a clear clinical meaning. By contrast, a standardized index that accurately reflects enteric health allows for bedside interpretability, rapid patient stratification, longitudinal tracking, and cross-study comparisons. Second, broad diversity metrics or ratios fail to capture the nuances of enteric health, whereas more sophisticated algorithms can account for confounding variables and the degree of importance that a particular feature (i.e., taxon) can contribute to the model. Finally, custom scoring can be disease-agnostic, meaning that a single standard health index can potentially flag dysbiosis even for conditions whose bacterial markers are not known.^161^ Thus, having a robust, rationally designed, and well-validated model could aid caregivers and researchers in finding correlations and causality between the composition and function of the gut microbiome and disease states. In this section, we focus our discussion on efforts to design objective metrics for dysbiosis, either for research purposes or as assays for routine clinical care.

### Diversity and richness as health indicators

Diversity-based metrics remain one of the most widely used and accessible proxies for assessing gut microbial health. Indeed, a central distinction between eubiosis and dysbiosis is the level of ecological diversity: eubiotic individuals typically harbor a richly diverse and stable microbial ecosystem, whereas dysbiosis is often marked by reduced diversity and compositional instability.^162^ Notably, α-diversity at baseline has been associated with favorable clinical outcomes in ICI therapies.^5,147^ Whereas higher body-mass index, weight, and blood pressure are generally associated with reduced diversity, increased gut microbial richness has been associated with regular physical activity and high intake of fruits and vegetables — factors generally attributed to good health.^163^ Reduced diversity has also been documented across a broad spectrum of diseases, including diarrhea,^164^ metabolic syndrome, obesity,^165^ and IBD.^166^

However, diversity readouts and their correlations with treatment outcomes or disease states can vary depending on cohort size and technical artifacts.^167^ Moreover, diversity alone cannot distinguish between beneficial and pathogenic communities, nor can it capture functional capacity or ecological dynamics. Thus, although diversity metrics have traditionally served as convenient proxies for gut health, the field is increasingly shifting toward more comprehensive and mechanistically informative approaches.

### Enterotypes and community typologies

Enterotypes were an early attempt to simplify gut microbial data by grouping microbial communities on the basis of the predominance of specific taxa. The concept defines clusters typically dominated by genera like *Bacteroides*, *Prevotella*, and *Ruminococcus*, although subsequent research has sometimes expanded or refined these categories.^168,169^ They can help stratify populations on the basis of broad gut microbiota composition, facilitating investigations into microbiota-driven disease mechanisms and therapeutic interventions. They also simplify the complexity of the gut microbiota into classifiers that can potentially help researchers and clinicians better understand the microbiome’s relationship with diet, metabolism, health, and disease.^170^

Several studies have illustrated the practical usefulness of enterotypes. In CRC, distinct signatures within enterotypes suggest potential diagnostic applications and personalized therapeutic strategies.^171^ They have also proven relevant in metabolic disorders like type 2 diabetes, in which Bacteroides-dominant enterotypes are correlated with higher metabolic risk profiles compared to *Prevotella*-dominant types, enabling targeted preventive or therapeutic interventions.^172,173^ In addition, enterotypes can predict individual responses to probiotics, with metabolic improvements like reductions in blood glucose or triglycerides being enterotype-dependent.^174^

However, enterotypes are subject to several controversies and challenges. A central debate revolves around whether gut microbiota form discrete clusters or exist along continuous “enterogradients”.^169^ In addition, methodological sensitivity impacts enterotype identification, with variations in clustering techniques, distance metrics, and sequencing methods leading to inconsistent results.^169,175^ Their clinical applicability is further challenged by emerging evidence that enterotypes exhibit variability over time, which is shaped by factors like diet, disease, and environmental changes.^176^ Moreover, diet and geography significantly influence enterotype distributions, underscoring the importance of considering these variables when interpreting enterotype-related findings.^169^ Finally, the number of enterotypes remains contentious, with different studies reporting 2–4 clusters,^177,178^ depending on the methods used and the populations studied. These inconsistencies and the absence of standardized protocols complicate cross-study comparisons and interpretations, limiting the broad applicability of enterotype research.

### Microbial guilds and functional groups

Originally an ecological concept, guilds are groups of species that use similar resources in similar ways and contribute to the same ecological function.^179,180^ Microbiologists have adapted this idea by defining a guild as a set of bacteria that rise and fall together in abundance and collaborate to deliver the same ecological function.^180,181^ These co-abundant taxa may originate from diverse phylogenies, but they share overlapping niche requirements and biological capabilities.^87,146,181–183^ This perspective highlights how microbial groups interact within complex ecosystems in the broader community, providing a function-focused alternative to cataloging diverse taxonomies.^180,181^

Given the complexity of microbial interactions, the identification of guilds requires sophisticated analytical methods. One approach is co-abundance network analysis, which clusters microorganisms on the basis of shared abundance trends.^146,180–182,184,185^ Techniques such as hierarchical clustering and weighted correlation network analysis have already been used to classify different taxa into guilds.^182,185^ These methods have revealed interesting contrasts within the microbiome, like the “two competing guilds” architecture, in which a health-associated foundation guild counterbalances a pathological guild enriched in virulence factors and antibiotic-resistance genes.^146,182,184,185^ The foundation guild typically contains butyrate-producing and mucosa-protective taxa such as *Faecalibacterium* spp., *Akkermansia* spp., and members of the *Lachnospiraceae* and *Oscillospiraceae* families.^87,146,182,185^ Context-specific variants of this beneficial guild have also been documented, such as the “fecal guild” expanding γδ intra-epithelial lymphocytes and a “small-intestinal guild” dominated by *Lactobacillus*, *Enterococcus*, and *Faecalibaculum*.^186^ By contrast, the pathological guild aggregates facultative or obligate anaerobes, such as *Enterocloster* and *Hungatella hathewayi*, pro-inflammatory oral taxa, and enterobacteria, whose outgrowth accompanies chronic inflammation, metabolic syndrome, and cancer progression.^4,184,187,188^ Intriguingly, a third metabolically defined guild composed of facultative anaerobes that exploit respiratory electron acceptors — such as oxygen — often surges during dysbiosis and serves as a broader ecological marker of disturbance.^17^ Identifying these guilds can help explain how shifts in collective metabolic capacity, rather than single-taxon perturbations, translate into host-level phenotypes and disease trajectories.

However, we still lack a rigorous and universally accepted framework for defining and quantifying guild units. Some criteria are so broad that they blur ecological meaning, whereas others are so restrictive that they exclude potentially significant taxa.^179^ Closing this gap will require systematic mapping of the metabolic pathways that distinguish one guild from another, whether fiber digestion and SCFA biosynthesis in health-promoting consortia or virulence and antibiotic-resistance pathways in pathogenic groupings.^146^

Equally vital are well-designed longitudinal studies that track how guilds — and the gut microbiota in general — evolve during ICI therapy. Temporal stability of certain beneficial functions or taxa has emerged as a consistent feature for durable responses.^189^ Responders tend to be enriched in *Clostridia*, particularly Lachnospiraceae, with SCFA-producing and fiber-degrading capacities, and pathways enriched in polyamine synthesis.^189,190^ Remarkably, one study also identified flagellin-related genes within these stable *Clostridia*, whose derived peptides structurally mimicked tumor-associated antigens and elicited cross-reactive CD8^+^ T-cell responses, providing a mechanistic link between microbial persistence and antitumor immunity.^189^ Another study found that postbiotics derived from *Lactobacillus paracasei* could upregulate HLA class I expression on cancer cells by engaging the NLRC5 protein (a major regulator of HLA expression), thus leading to tumor antigen-specific T-cell activation and synergistic anticancer effects with PD-1 blockade therapy.^191^ Importantly, the microbial composition of patients receiving anti-PD-1 therapy is not static, and taxa that appear agnostic toward treatment outcomes at baseline may become deleterious at later timepoints.^190^ Indeed, ~9–10 months after treatment initiation, non-responders frequently display an expansion of Gram-negative taxa, accompanied by elevated neutrophil-to-lymphocyte ratios and signatures of LPS-driven inflammation.^132^ Collectively, these findings indicate that longitudinal stability, both in microbial presence and immune-relevant function, may reveal more information for predicting ICI efficacy than baseline diversity alone.

Epistatic effects between bacteria have also been shown to influence their function, which may translate into clinical consequences.^24,192^ These interbacterial relationships may redefine our understanding of microbiome–immune–tumor interactions and warrant further investigation to clarify their clinical relevance. Only by pairing precise functional annotation with temporal dynamics can we transform the guild framework from a descriptive shorthand into a predictive tool for personalized interventions.

### ML- and multi-omics-driven biomarker discovery

In recent years, the pursuit of reliable biomarkers for gut dysbiosis has shifted markedly toward ML- and multi-omics-driven strategies. Several dysbiosis indices have already been proposed in the context of various conditions, including cirrhosis,^193^ gastrointestinal maladies,^194–196^ gout,^197^ heart disease,^198,199^ and cancer.^200,201^ Other researchers have attempted to develop a more generalized approach,^161,188,202^ and at least one study has integrated metagenomics and proteomics to define a functional, rather than compositional, metric for dysbiosis.^203^ Wei et al.^204^ have written the most recent review of the variety of algorithms and formulae being used to calculate dysbiosis metrics, and we encourage readers to consult their article for further insights.

Examples of some early models for calculating a dysbiosis score relied primarily on ratios of bacteria of interest in a disease-specific context. For example, the Microbial Dysbiosis Index was developed in the context of Crohn’s disease (CD) and is calculated as the logarithmic ratio of bacteria that increase in CD patients to those that decrease.^205^ This model inspired the development of the Microbial Community Polarization index in a study of CRC carcinogenesis.^201^ Another early metric is the GA-map dysbiosis test, which uses a probe-based assay and a proprietary algorithm to identify and rank the degree of dysbiosis in IBD patients against a reference “normobiotic” microbiome.^194^

Recently, more sophisticated models validated against hundreds to thousands of individuals have emerged. Jaeyun Sung developed the Gut Microbiome Health Index (GMHI).^161^ His group examined the metagenomic data from 4347 microbiomes to develop a mathematical formula based on the prevalence and relative abundance of 50 key bacterial species, 43 of which (86%) were labeled as “health scarce” species. This strategy enabled the GMHI to discriminate between healthy and diseased individuals with an accuracy of 73.7%. The GMHI was later upgraded on the basis of a larger data set of 8059 metagenomes from 54 studies and renamed the Gut Microbiome Wellness Index 2 (GMWI2).^202^ In addition, the GMWI2 used a strategy that weighted the contribution of each taxon on the basis of its strength of association with dysbiosis rather than assuming that all taxa contribute equally. These differences enabled GMWI2 to outperform GMHI and achieve a higher dysbiosis classification accuracy of up to 90%.^202^

The HACK (Health-Associated Core Keystone) index takes a different approach. Rather than determining which microbial species are associated with disease, Goel et al.^188^ aimed to identify the hallmarks of a eubiotic gut and concluded that subjects with deviations from this compositional benchmark are dysbiotic. They analyzed 45,424 adult metagenomes from 42 countries and identified 201 key taxa using three key parameters: their association with non-diseased subjects; stability over time (i.e., how prevalent they remained within longitudinal samples); and correlation with host health. The authors could then use the 17 top taxa in their ranked list to identify which individuals are dysbiotic, an approach that demonstrated similar or better performance than the GMWI2 or another published metric called the “dysbiotic score”.^195^ Notably, all 17 bacteria on this list — including *F. prausnitzii*, *Roseburia* spp., and *Alistipes shahii* — are obligate anaerobes and known butyrate producers.^206^

ML is crucial for analyzing such high-dimensional data. Unsupervised clustering and supervised models sift through thousands of microbial genes, metabolites, and proteins to find optimal marker panels. For example, large cancer microbiome consortia (such as the EU ONCOBIOME project^207^) have applied ML to define composite dysbiosis scores predictive of therapy response. The TOPOSCORE — one example developed by our lab (discussed below)^200^ — is designed to guide interventions and identify patients who might benefit from MCIs such as dietary changes, probiotics, live therapeutics, postbiotics, or FMT before or alongside immunotherapy.^208^ Although it requires prospective validation, the TOPOSCORE demonstrates how ML can integrate complex microbial signatures into a practical biomarker tool for clinical decision making.

ML-based models have demonstrated that dysbiosis in cancer resembles dysbiosis in the context of other chronic inflammatory conditions. This suggests that dysbiosis scoring may be broadly applicable across disease states, potentially eliminating the need for disease-specific models. In addition, instead of examining each biomarker individually, researchers are integrating metagenomics, metabolomics, proteomics, and immunological data to uncover multi-dimensional signatures of dysbiosis.

One compelling example is the metagenome-informed metaproteomics pipeline.^203^ This approach uses shotgun metagenomic sequencing to inform high-resolution mass spectrometry-based proteomics, enabling nearly complete identification of proteins in stool and distinguishing endogenous, microbial, and dietary proteins.^203^ Applying this pipeline to IBD patients led to the discovery of dozens of novel proteins (both host- and microbe-derived) that were altered in dysbiosis. Remarkably, certain combinations of these proteins could discriminate disease subtype and severity, outperforming fecal calprotectin (the current gold standard) in diagnostic accuracy. The analysis also revealed previously unrecognized human anti-microbial peptides upregulated in dysbiotic guts.^203^ These peptides and other host- and bacteria-derived proteins, such as haptoglobin, ppdK (a glycolytic enzyme), and type 1 glyceraldehyde-3-phosphate dehydrogenase, form a rich new class of biomarker candidates. By profiling the bacterial proteome, metaproteomics delineates the functional activities that are preserved or impaired in dysbiosis. This kind of multi-omic atlas of the gut ecosystem is revolutionizing biomarker discovery: rather than relying on hypothesis-driven markers, it enables data-driven identification of signature molecules that track gut health with high specificity.

These methods are only a few examples of the many models presented in the literature for determining whether a patient is dysbiotic. Nevertheless, fundamental questions remain. Are classical approaches based on relative abundance variations the most informative means of studying gut microbial composition? Given the numerous confounding factors in microbiome research and the challenges of linking compositional data to quantitative pathophysiological parameters,^209,210^ complementary strategies should be considered. These include quantifying bacterial loads or absolute species counts^211^ through methods such as 16S rRNA amplicon sequencing, flow cytometry, and ML-based analyses.^209,210,212,213^ Are deviations of the gut microbiota repertoire the cause or the consequence of the morbid state with its comedications? Finally, what are the underlying factors driving dysbiosis?

## Key factors contributing to cancer-associated dysbiosis

To delve deeper into these pivotal questions, we used our innovative TOPOSCORE metric.^200^ This ecology-centric approach identifies gut microbial species that are correlated with survival in cancer immunotherapy based on ICIs alone or in combination with chemotherapy in metastatic patients. TOPOSCORE is an individualized metric of dysbiosis, whereas guilds, alpha and beta diversity, and the other methods described above require population-level datasets to yield meaningful information. The TOPOSCORE demonstrates performance comparable to those of other models while still retaining clinical practicality, enabling longitudinal assessment of the impact of disease evolution or treatment and translating into a rapid, bedside-compatible qPCR test. Thus, it offers a user-friendly approach that can rapidly determine whether a patient has gut dysbiosis at any time during their treatment regimen.^200^

Using shotgun metagenomics and co-abundance network analysis based on the MetaPhlAn 4 (ChocoPhlAn 2021) pipeline,^214^ our lab identified two distinct and antagonistic microbial constellations, referred to as species interaction groups (SIGs), which provide valuable insights into the dynamics driving dysbiosis.

The SIG1 group is composed of 37 bacterial species frequently associated with worse overall survival following immunotherapy. It consists of two smaller clusters that were identified when the pre-constructed co-abundance network was partitioned by Ward-linkage hierarchical clustering. One cluster is composed of taxa resident primarily in the oral cavity, like *Veillonella* spp., *Streptococcus* spp., and *Bifidobacterium dentium*. The other cluster contains gut-resident obligate anaerobes known for their BA production, such as *Clostridium* spp., *Enterocloster* spp., and *H. hathewayi*. Functionally, these organisms converge on BA metabolism and pro-inflammatory signaling, with oral taxa introducing aerotolerant, inflammation-prone communities and *Enterocloster* and *Clostridium* spp. disrupting mucosal immune homing. This is notable because (as discussed above) we have previously demonstrated that species from the *Enterocloster* genus can bloom following cessation of antibiotics, leading to altered BA metabolism, reduced MAdCAM-1 expression, and exodus of enteric Tr17 cells to distant tumors.^4^ SIG1 bacteria also display functions related to fatty acid oxidation, succinate fermentation to butanoate, degradation of L-histidine and L-phenylalanine, and the biosynthesis of L-serine, L-lysine, and L-glycine.^200^

**Fig. 4:**
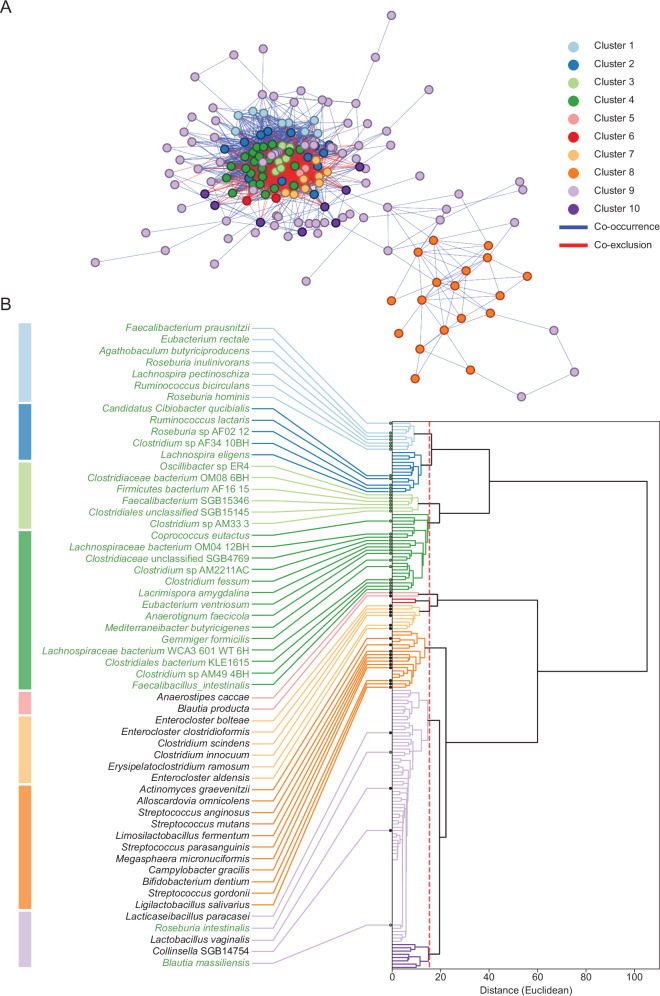
Expanded gut microbiome network and hierarchical clustering in a pan-cancer cohort. **a** Co-abundance network reconstructed with the workflow presented by Derosa et al.^24,201^ but applied to the microbiomes of all 955 cancer patients in our dataset (NSCLC, *n* = 556; RCC, *n* = 82; UC, *n* = 133; CRC, *n* = 182). Nodes (individual metagenomic species) are colored by cluster. Blue edges denote positive co-occurrence and red edges negative co-exclusion. **b** Dendrogram of the same species, with colored bars beside the dendrogram corresponding to the network clusters shown in **a**. Species labeled in green are immunotherapy-responsive SIG2 bacteria, whereas those in black are resistance-associated SIG1 bacteria. The dashed red line marks the distance threshold that segregates the 10 major clusters.

By contrast, the SIG2 group contains 45 bacterial species that are often associated with a better treatment prognosis (Fig. 4). All bacteria in this group are gut-resident obligate anaerobes and butyrate producers that stabilize barrier function and promote T-cell tolerance, such as *Faecalibacterium* spp.,^215^ *E. rectale*,^216^ *Coprococcus comes*,^217^ *Roseburia inulinivorans*,^218^ and *Oscillibacter* sp. ER4.^219^ Butyrate modulates peripheral immune tolerance,^220^ acts as a key resource for colonocytes,^126^ improves gut barrier integrity,^129^ and helps maintain the O_2_-deprived environment in the gut lumen necessary for the growth of anaerobic bacteria.^130,221^ The SIG2 bacterial consortium also has functions associated with tRNA processing, peptidoglycan biosynthesis, purine nucleoside and mannan–stachyose degradation, the GABA shunt, L-glutamate and L-glutamine biosynthesis, the polyamine pathway (including L-ornithine and L-arginine biosynthesis), and sulfur oxidation.^200^

The fecal prevalence of these species (evaluated using MetaPhlAn 4, ChocoPhlAn 2021^214^) is used to calculate and assign an individual SIG score ($S$). This value is defined as:

$$S=\frac{1}{2}\left(\frac{{N}_{{SIG}2}}{45}-\frac{{N}_{{SIG}1}}{37}+1\right)$$

Where *N*_*SIG1*_ and *N*_*SIG2*_ are the numbers of unique SIG1 and SIG2 taxa detectable in a fecal sample, respectively. Patients who are evaluated as *S* ≤ 0.535 are SIG1+ (dysbiotic), whereas those with a score *S* ≥ 0.791 are SIG2+ (eubiotic). “Gray zone” patients — i.e., those not immediately assigned to SIG1+ or SIG2+ — are further stratified on the basis of the prevalence and relative abundance of *A. muciniphila* SGB9226. Our lab and others have demonstrated the antitumor effects of *A. muciniphila*,^13,222,223^ but we have also shown that there is a non-linear, trichotomic relationship between the relative abundance of *A. muciniphila* SGB9226 and patient overall survival.^3^ Gray zone patients who lack or have an overabundance (> 4.799%) of *A. muciniphila* are considered SIG1+, whereas others with “normal” levels are considered SIG2+. Our TOPOSCORE formula has been validated across hundreds of patients and multiple malignancies, demonstrating a predictive accuracy of 69.8%.^200^ In addition, a distilled variant incorporating 21 bacterial taxa — five SIG1 and 17 SIG2 — can better predict the survival of CRC and melanoma patients. The TOPOSCORE algorithm is implemented in R and freely available on GitHub (http://github.com/valerioiebba/TOPOSCORE), with an interactive Shiny application accessible via the ONCOBIOME Atlas (https://oncobiome-atlas.shinyapps.io/TOPOSCORE/).

**Fig. 5:**
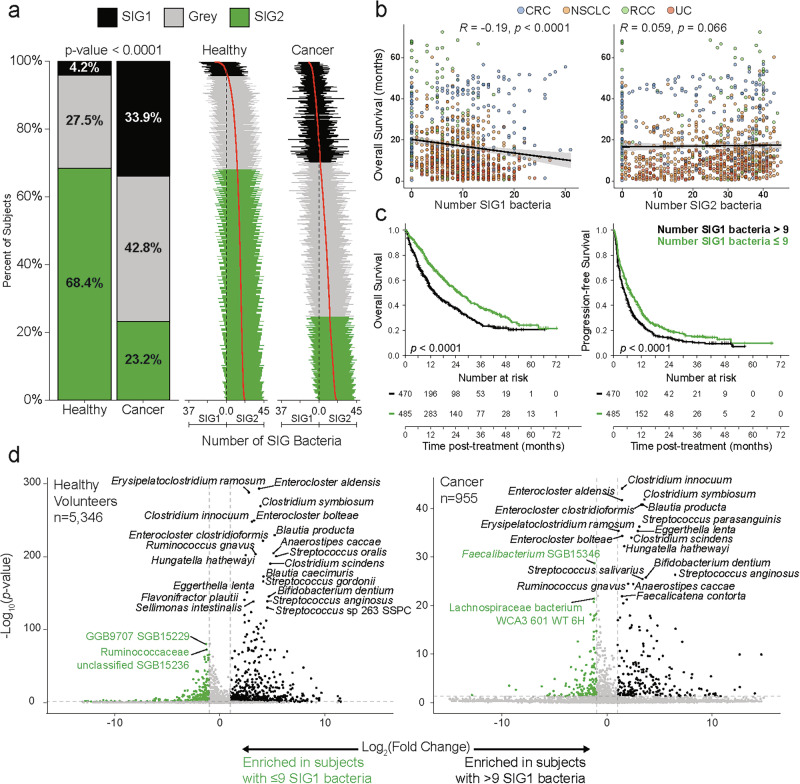
SIG1 enrichment is a microbiome signal of dysbiosis and poor clinical outcome across cancers. TOPOSCORE profiling of 5346 healthy volunteers and 955 cancer patients confirmed that an excess of SIG1 taxa — bacteria linked to ICI resistance — marks a shift from eubiosis to dysbiosis and carries systemic consequences. **a** Cancer patients are significantly more likely than healthy subjects to have a dysbiotic (SIG1) gut or be classified as Gray (Fisher’s exact test, *P* < 0.0001). The right panel shows that in healthy individuals, the balance is typically skewed toward having more SIG2 bacteria, whereas in cancer patients, the balance shifts toward a dominance of SIG1 bacteria, highlighting an ecological re-organization. **b** Across CRC, NSCLC, RCC, and UC patients, the number of SIG1 species is negatively correlated with overall survival (Spearman, R = –0.19, *P* < 0.0001). By contrast, SIG2 counts show no meaningful association with survival (R = 0.059, *P* = 0.066). **c** Using a median threshold of *n* = 9 SIG1 species, patients above the cut-off experience markedly shorter overall and progression-free survival (log-rank *P* < 0.0001), further underscoring the clinical relevance of SIG1. **d** Volcano plots reveal that SIG1-high healthy volunteers (left) and cancer patients (right) are consistently enriched for pro-inflammatory (*E. ramosum*), immunosuppressive (*Enterocloster* spp., *H. hathewayi*, *Clostridium* spp.), or oral taxa, suggesting that these genera are the principal ecological drivers of dysbiosis. SIG, Species interacting group.

To better understand the factors driving dysbiosis, as defined by the TOPOSCORE, we retrospectively analyzed metagenomics data from 5346 healthy volunteers obtained from publicly available sources^224^ and 955 cancer patients^24^ diagnosed with non-small cell lung cancer (NSCLC, *n* = 556), RCC (*n* = 82), and urothelial carcinoma (UC, *n* = 133), along with CRC patients (*n* = 182) from the AtezoTRIBE study (NCT03721653).^225–227^ In healthy individuals, most stool samples fell into the eubiotic SIG2 category (~68%), whereas only ~4% mapped to the adverse SIG1 group (Fig. 5a, left plot). Cancer patients showed the opposite pattern: 34% of subjects were SIG1, whereas ~23% were SIG2. The mirror-histogram plots on the right further highlight a clear increase in the number of SIG1 taxa per metagenomic sample among the cancer patients, indicating a significant re-structuring of the microbiomes. We can also see that there is a clear consequence when we examine correlations between the number of prevalent SIG1 bacteria in cancer patients and overall survival (Fig. 5b). Each dot represents an individual microbiome at baseline, with CRC, NSCLC, RCC, or UC patients represented in blue, orange, green, and brown, respectively. Overall survival decreases as the SIG1 count increases (Simpson R = –0.19, *P* < 0.0001), whereas the analogous analysis for SIG2 shows no significant correlation (R = 0.06, *P* = 0.066). Thus, it is the accumulation of SIG1 organisms, rather than the prevalence of SIG2, that predicts poor treatment outcomes following immunostimulation with ICI.

Moreover, clear patterns emerge when centered log-ratio-normalized abundances are plotted as a heatmap (Supplementary information, Figs. S1, S2). Both healthy individuals and cancer patients classified as SIG2+ demonstrate a diverse representation of SIG2 bacteria with very low levels of SIG1 bacteria. Notably, some of the SIG1 bacteria are not completely absent but rather appear to maintain homeostasis at low levels. Individuals classified as SIG1+ appear to fall into at least two distinct groups based on the identity of the SIG1 bacteria that dominate, which we herein define as Type 1 and Type 2 compositional dysbiosis. Type 1 dysbiosis is characterized as a modest loss of SIG2 bacteria with evidence of oral species spillover, as exemplified by the presence of aerotolerant genera like *Veillonella* and *Streptococcus*. Type 2 dysbiosis presents as a near complete collapse of the SIG2 consortium with concomitant over-representation of gut-resident, obligate anaerobes in the SIG1 group (e.g., *Enterocloster* and *Clostridium* genera). These two dysbiosis trajectories highlight distinct ecological failures: Type I reflects a loss of colonization resistance against aerotolerant invaders, whereas Type II reflects the collapse of fermentative consortia critical for SCFA production and barrier integrity.

We then examined how increased numbers of SIG1 bacteria impact patient survival (Fig. 5c). Using the median value of nine SIG1 taxa as a threshold, we showed that patients with a higher SIG1 burden (black curves) experienced markedly worse overall and progression-free survival; median time to progression was reduced by almost 2 years compared with that of patients harboring nine or fewer SIG1 bacteria at baseline (green curves). We next investigated which microbes drive the signal (Fig. 5d). The volcano plot of cancer samples identifies *Clostridium innocuum*, *Enterocloster clostridioformis*, and *Erysipelatoclostridium ramosum* as among the taxa most strongly enriched in high-SIG1 carriers, whereas Lachnospiraceae bacterium WCA3 or *Faecalibacterium* SGB15346 predominate in the low-SIG1 subset. Notably, a similar result was found among SIG1+ healthy volunteers, who also had higher abundances of species from the *Enterocloster* and *Clostridium* genera. Taken together, these results position SIG1 enrichment as a hallmark of cancer-associated dysbiosis, tie it to shorter survival, and pinpoint candidate organisms responsible for the compositional shift.

## Oncobiome network: establishing an international clinical framework for MCIs

The concept of “gut health” as a clinical biomarker is rapidly gaining traction in oncology, transitioning from a theoretical construct to an actionable tool for patient stratification and treatment guidance. We are now beginning to recognize that eubiosis, rather than being defined by a fixed catalog of taxa, depends on gut microbial resistance (the ability to withstand insult) and resilience (the capacity to recover after disturbance).^189,228^ Dysbiosis can be framed as a disruption of these dynamics rather than a simple loss of diversity, as exemplified by longitudinal studies of patients receiving anti-PD-1 therapy.^189^ Stable taxa and functions are enriched in therapy responders across multiple cohorts, further supporting the idea that a resilient microbiome underpins effective ICI therapies.^190,229^ In this context, metabolic independence — the capacity to synthesize essential cofactors, amino acids, and nucleotides — has emerged as a key ecological trait that promotes enteric health.^228,230^

Clinical evidence from FMT trials, which are being investigated to correct dysbiosis, appears to illustrate this ecological perspective. These studies demonstrated that recipients’ gut microbiota diverged from baseline after FMT and matched those of their respective donors.^229,231,232^ Moreover, their compositions remained stably divergent from baseline over the 7–65-day follow-up, demonstrating that donor-specific engraftment can persist for weeks, providing there is no influence from external factors.^231^ Indeed, one study reported that a patient who received antibiotics 11 weeks after their first FMT required a second FMT almost a year later to restore donor-like microbiota, thus highlighting the fragility of engraftment under external pressures.^232^ The persistence of engraftment was supported by follow-up studies showing recurrence of dysbiosis and clinical relapse after antibiotic use, which was also subsequently corrected with a second FMT.^233^

Together, these studies indicate that FMT is not a static transfer of taxa but a dynamic intervention whose success depends on the recipient’s resistance and resilience, the donor’s microbial repertoire, and external factors. However, the duration of these effects is still not understood. The longest follow-up among FMT trials for cancer patients has been 3 months.^229,231,232,234^ Evidently, this short timeframe has been sufficient to reset the holosystem and prime it for improved responses to ICI (mouse models have demonstrated that FMT can restore the expression of ileal MAdCAM-1^4^), but long-term effects still need to be determined. Furthermore, there is an assumption that successful engraftment following FMT is required for clinical success. This has yet to be conclusively demonstrated. In fact, a metagenomics analysis of 316 FMTs indicated that clinical success does not rely on successful colonization, microbial displacement, or bacterial reinstatement.^230^ Building on these ecological and translational insights, large-scale initiatives are now seeking to operationalize this knowledge within a clinical framework.

ONCOBIOME is a large-scale international initiative that seeks to manipulate the gut microbiota to improve outcomes in cancer immunotherapy.^207^ It was launched in 2019 and brings together academic institutions, research centers, and private industry partners across eight countries. ONCOBIOME views dysbiosis as a treatable condition, not just a biomarker. Thus, it is built around two central objectives: first, to define Gut OncoMicrobiome Signatures (GOMs)^43^ and translate them into diagnostics and clinically actionable interventions; and second, to translate this knowledge into innovative and effective MCIs. To achieve these goals, ONCOBIOME emphasizes broad multidisciplinary collaboration to standardize metagenomic protocols, integrate microbiome, immune, and clinical datasets, and apply GOMs to guide precision immunotherapy. The TOPOSCORE is one deliverable that was developed and validated by this project.^200,207^ Through these efforts, ONCOBIOME has laid the scientific and infrastructural groundwork for microbiome-informed precision oncology.

One initiative within the broader ONCOBIOME framework is IMMUNOLIFE2 (NCT07001618), a randomized Phase II trial of FMT to restore ICI sensitivity in cancer patients who have become resistant owing to antibiotics. Pioneering studies in mice and melanoma patients have already demonstrated its safety and efficacy in improving anti-PD1 therapy,^13,229,231,232,235^ and additional studies and meta-analyses are investigating its mechanism of action.^230,234^ IMMUNOLIFE2 targets advanced lung cancer patients who previously received antibiotics and subsequently demonstrated primary resistance to PD-1 blockade therapy. The goal is to administer MaaT033 — an oral formulation of standardized, pooled-donor FMT capsules — in combination with anti-PD-1 therapy and compare outcomes against a standard-of-care control arm to determine whether FMT can restore immunotherapy responsiveness in patients who would otherwise have poor outcomes following antibiotic-induced resistance. The success of these studies could support the implementation of antibiotic stewardship recommendations^236^ and pave the way for routine microbiota restoration (via FMT or next-generation MCIs) before re-challenging cancer patients with immunotherapy.

Launched in 2025, the Seerave Global OncoBiome Atlas expands the ONCOBIOME initiative from a European-based research consortium into a globally integrated clinical and translational platform (https://oncobiomeatlas.org). The Atlas is designed to be an interactive, longitudinal resource that links microbiome profiles, comedication records, and immunotherapy outcomes across international cancer cohorts. Its core mission is to systematically track the use of microbiome-modulating drugs to better understand their real-world effects on the gut ecosystem and treatment efficacy. Thus, whereas ONCOBIOME focuses on defining GOMs and developing MCIs, the Seerave Atlas provides the global infrastructure necessary to validate, refine, and implement those interventions in routine oncology care.

Together, ONCOBIOME and the Seerave Global OncoBiome Atlas create a platform for the prospective validation of microbiome-based tools, like the TOPOSCORE, which will soon be hosted on the Seerave website. The free accessibility of this tool enables clinicians and researchers to investigate the clinical relevance of gut microbial profiles, particularly the number and identity of SIG1 bacteria, for predicting immunotherapy outcomes (Fig. 5). MCIs are increasingly being evaluated in clinical trials worldwide.^236^ Now, this partnership will enable investigators to apply tools like the TOPOSCORE prospectively across multiple cancer types to validate their efficacy, better stratify patients, and refine therapeutic strategies.

## Conclusion

The gut microbiome is a central determinant of immunotherapy efficacy, particularly in patients treated with ICIs. With this understanding, more nuanced models for predicting whether a patient is dysbiotic — and thus less likely to respond to treatment — are incorporating fecal, serological, and taxonomic biomarkers and are continually being developed. Exact definitions for dysbiosis and eubiosis remain elusive. Nonetheless, translational efforts aim to operationalize these findings through dedicated diagnostics, longitudinal monitoring, and targeted interventions, and ongoing clinical trials are directly testing whether MCIs can restore ICI sensitivity in patients.^88,236^ Importantly, these efforts can be translated to other immunotherapies. For example, CAR T-cells,^237,238^ hematopoietic stem cell transplantation,^239^ and vaccines^244^ are all affected by gut microbial composition. Although individual taxa vary across cohorts, three ecological themes recur: preservation of SCFA-producing commensals, containment of oral and BA-modulating pathobionts, and maintenance of barrier integrity. Framing dysbiosis through these shared functions provides a mechanistic bridge across otherwise disparate taxonomic associations. Moving forward, the integration of microbiome metrics into precision oncology represents a logical and necessary extension of current treatment plans.

## Supplementary information

Supplementary information, Fig. S1

Supplementary information, Fig. S2
