# Supplementary material for: Gut dysbiosis in oncology: a risk factor for immunoresistance
Source: Cell Res. 2026 Jan 14;36(2):103–20. doi: 10.1038/s41422-025-01212-6 (PMC12847903; doi:10.1038/s41422-025-01212-6)
Supplement: Supplementary file 1 — Supplementary information, Fig. S1 [file 41422_2025_1212_MOESM1_ESM.pdf]

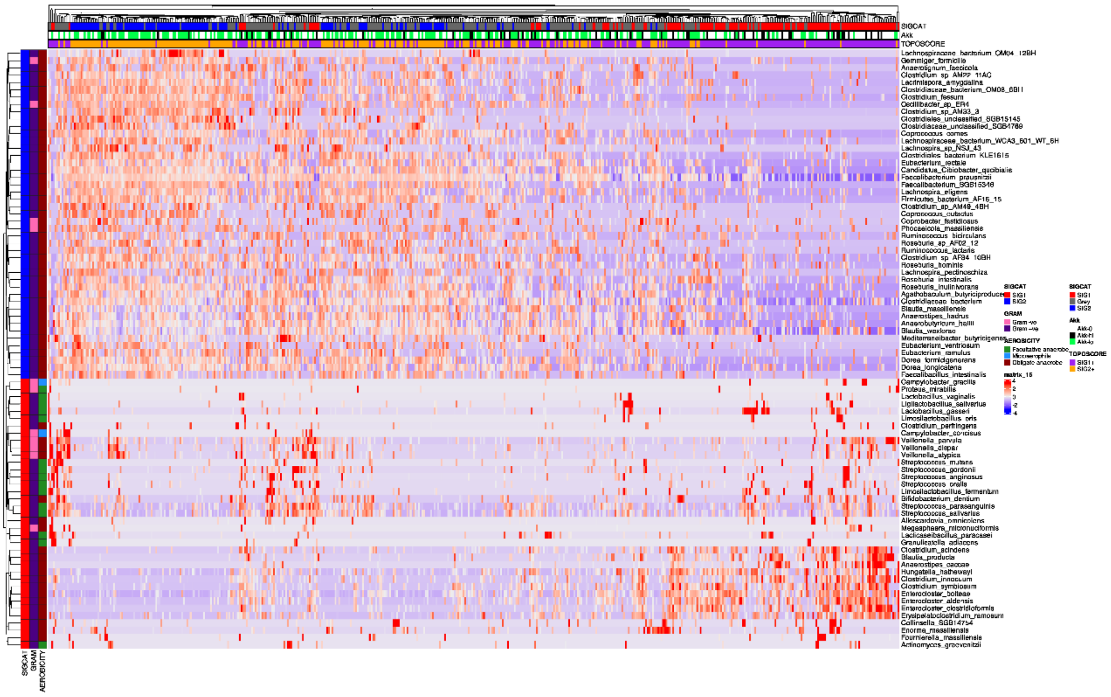

Supplementary information, Fig. S1 Landscape of SIG1 and SIG2 bacterial abundances across 955 cancer microbiomes. Heatmap depicting the log-transformed, z-score-normalized relative abundances of 82 signature gut taxa (SIG1, n=45; SIG2, n=37) in fecal metagenomes from 955 cancer patients. Each column represents one individual — non-small-cell lung cancer (NSCLC, n=556), renal cell carcinoma (RCC, n=82), urothelial carcinoma (UC, n=133) and colorectal cancer (CRC, n=182) — color-coded in the top annotation bar. Rows correspond to bacterial species, ordered by hierarchical clustering. Red cells denote taxa with higher abundances, whereas blue cells indicate depletion. Notably, two key dysbiotic patterns emerge: Type I dysbiosis (far left), characterized by a modest loss of the SIG2 consortium together increased abundances of SIG1 oral commensals (e.g., *Streptococcus* species.), and Type II dysbiosis (far right), distinguished by near-complete collapse of SIG2 bacteria and simultaneous blooms of *Enterocloster* and *Clostridium* species. Akk, *A. muciniphila*; SIGCAT, SIG category; TOPOSCORE, Topological score.
