# Supplementary material for: Gut dysbiosis in oncology: a risk factor for immunoresistance
Source: Cell Res. 2026 Jan 14;36(2):103–20. doi: 10.1038/s41422-025-01212-6 (PMC12847903; doi:10.1038/s41422-025-01212-6)
Supplement: Supplementary file 2 — Supplementary information, Fig. S2 [file 41422_2025_1212_MOESM2_ESM.pdf]

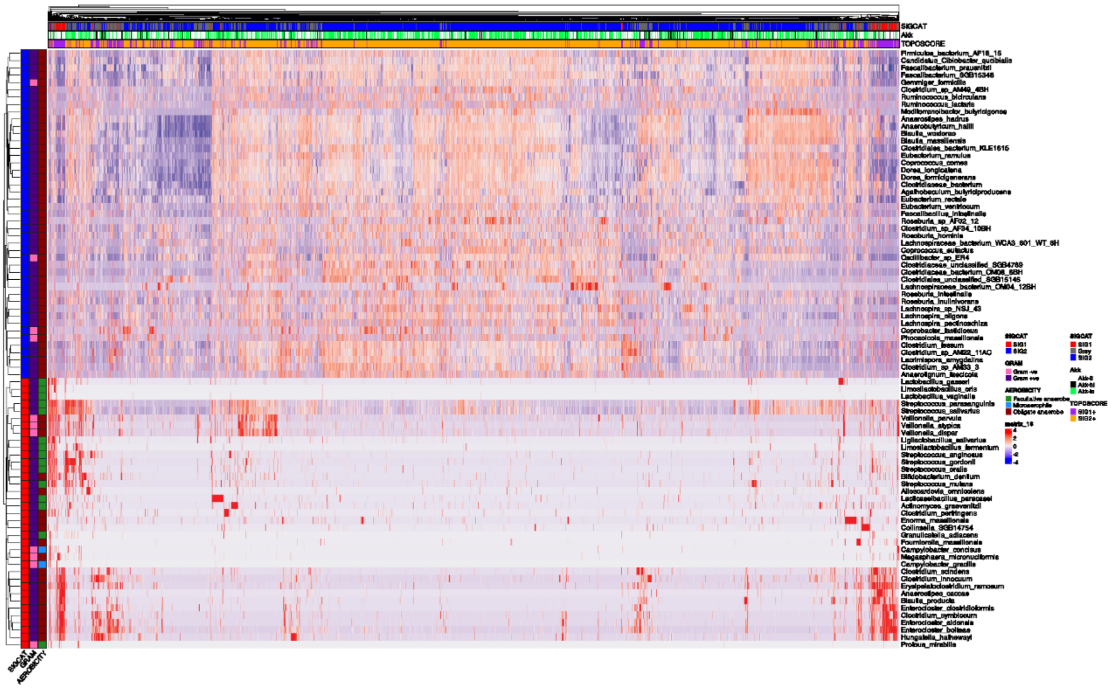

Supplementary information, Fig. S2 Landscape of SIG1 and SIG2 bacterial abundances across 5,346 healthy volunteer microbiomes. Same as Supplementary information, Fig. S1 but for 5,346 healthy volunteers. Like with the cancer cohort, two notable dysbiosis types can be observed in a minority of subjects. Type I dysbiosis is marked as an increase in abundance of oral taxa, while Type II presents as an overabundance of *Enterocloster* and *Clostridium* spp. at the expense of SIG2 bacteria. These data illustrate that the dysbiotic patterns prominent in cancer patients also exist in healthy participants, underscoring the utility of the SIG1/SIG2 framework for discriminating between eubiotic and dysbiotic microbiomes across disease states. Akk, *A. muciniphila*; SIGCAT, SIG category; TOPOSCORE, Topological score.
